# Fasting Plasma Ketone Bodies Are Associated with NT-proBNP: A Potential Mechanism to Provide Fuel for the Failing Heart

**DOI:** 10.3390/jcm13061541

**Published:** 2024-03-07

**Authors:** Constantin L. Palm, Irina Shalaurova, Margery A. Connelly, Stephan J. L. Bakker, Berend Daan Westenbrink, Robin P. F. Dullaart

**Affiliations:** 1 Department of Cardiology, University of Groningen and University Medical Center Groningen, 9700 RB Groningen, The Netherlands; b.d.westenbrink@umcg.nl; 2 Labcorp, Morrisville, NC 27560, USA; shalaui@labcorp.com (I.S.); connem5@labcorp.com (M.A.C.); 3 Department of Internal Medicine, Division of Nephrology, University of Groningen and University Medical Center Groningen, 9700 RB Groningen, The Netherlands; s.j.l.bakker@umcg.nl; 4 Department of Internal Medicine, Division of Endocrinology, University of Groningen and University Medical Center Groningen, 9700 RB Groningen, The Netherlands; dull.fam@12move.nl

**Keywords:** cardiology, epidemiology, heart failure, ketone bodies, N-terminal pro-B-type natriuretic peptide

## Abstract

**Background:** Heart failure (HF) features a shift in metabolism towards enhanced utilization of ketone bodies. While elevations in plasma natriuretic peptides represent a biochemical hallmark of HF, natriuretic peptides may promote lipolysis, thereby contributing to fatty acid availability for ketogenesis. **Methods:** We cross-sectionally tested to what extent fasting plasma total ketone bodies (measured using nuclear magnetic resonance spectroscopy) are associated with N-terminal pro-BNP (NT-proBNP; electrochemiluminescent sandwich immunoassay) in individuals with and without HF. **Results:** Among 6217 participants from the Prevention of REnal and Vascular ENd-stage Disease (PREVEND) study, 203 were identified with HF. NT-proBNP was four-fold and total ketone bodies were 25% higher in HF participants (each *p* < 0.001). In both participants with and without HF, total ketone body levels correlated with NT-proBNP (r = 0.116 and 0.185, respectively; *p* < 0.001). In multivariable linear regression analysis adjusted for relevant covariates, total ketone bodies remained associated with NT-proBNP in the whole cohort (std β = 0.08, *p* < 0.001), without a difference in participants with and without HF (*p* interaction: 0.52). **Conclusion:** This general population-based study reveals an independent association of fasting total body ketone bodies with plasma NT-proBNP. Our findings suggest that a metabolic defense mechanism could be operative, providing the myocardium with ketone bodies to meet its energy demands.

## 1. Introduction

Heart failure (HF) confers a major cardiovascular disease (CVD) burden worldwide, resulting in a considerable demand on healthcare resources [1,2,3,4]. Although the prevalence of HF varies widely across countries, it markedly increases with age [3,4]. For these reasons, continuous efforts are necessary to better understand the complex and multifaceted metabolic abnormalities that occur during its development.

The human body shows remarkable metabolic flexibility and is able to utilize different substrates for energy production depending on their availability [5]. Ketogenesis is the metabolic process whereby fatty acids are converted into ketone bodies, i.e., β-hydroxybutyrate, acetoacetate and acetone, which takes place in hepatocyte mitochondria [5,6]. Fatty acid β-oxidation leads to the production of acetyl-CoA, which can be utilized as a substrate for the tricarboxylic acid (TCA or Krebs) cycle or can be converted to acetoacetyl-CoA and eventually to acetoacetate, from which β-hydroxybutyrate is derived [5]. Acetone, the least abundant ketone body, is the spontaneous breakdown product of acetoacetate. Lipolysis in adipose tissue results in enhanced delivery of fatty acids to the liver, making it imperative for ketogenesis, which in turn is stimulated during prolonged fasting, (relative) insulin deficiency and neurohumoral stress [5,6].

It is well recognized that circulating ketone bodies are elevated in both chronic and acute HF [7,8]. Indeed, exhaled acetone may represent a biomarker of HF severity [9]. Moreover, in a general population cohort, plasma β-hydroxybutyrate was found to predict incident HF, mainly HF with reduced ejection fraction (HFrEF) in women [10]. Alterations in substrate metabolism are likely to play a role in contractile dysfunction, which is characteristic of HF. Earlier data have suggested that there is impaired fatty acid oxidation and increased glycolysis and glucose oxidation in more advanced stages of HF [11]. More recently, it has been shown that the failing heart increasingly uses β-hydroxybutyrate for substrate metabolism, as demonstrated in mice [12]. Interestingly in humans, the heart consumes ketone bodies directly proportional to their plasma concentration levels, with more ketone bodies being consumed in HF [13]. Combined, these data are consistent with the possibility that the increase in circulating ketone bodies in HF patients may provide an adaptive metabolic defense mechanism for the failing heart [12,13,14].

Measurement of circulating levels of natriuretic peptides (NPs), in particular, B-type natriuretic peptide (BNP) and N-terminal pro-BNP (NT-proBNP), represents a valuable biochemical tool for establishing a diagnosis of HF, both in HFrEF and in HF with preserved ejection fraction (HFpEF) [1,3,15,16]. Absence of NP elevations essentially rules out a diagnosis of HF [1], while NP measurement could be used as a guide for HF treatment and a surrogate end-point for HF intervention trials [16,17]. Despite this, metabolic actions of NPs have received less attention. In addition to key roles in regulating sodium and fluid homeostasis, NPs are able to stimulate lipolysis in human adipocytes in vitro and promote lipid mobilization in vivo [18,19,20]. Abundant atrial natriuretic peptide (ANP) receptor expression has been found in isolated human fat cells with ANP and BNP exerting potent lipolytic actions, whereas microdialysis experiments have demonstrated a strong lipolytic effect of ANP in subcutaneous adipose tissue from lean and obese individuals [21,22]. These effects of NPs seem unrelated to catecholamine-mediated stimulation of lipolysis [21,22], suggesting that NPs exert their lipolytic effects independent of the stress response observed in HF [14,20]. To translate these pathophysiological findings [18,19,20,21,22] to the clinical setting, it is relevant to discern relationships between circulating NPs and ketone bodies in individuals with and without HF. However, aside from a study that showed a positive correlation of plasma acetone with NT-proBNP in 79 patients hospitalized for acute heart failure [23], there is only one report that evaluated the relationship between NPs and the levels of ketone bodies [24]. In that study, among 1030 patients undergoing cardiac catheterization, BNP was positively associated with total ketone bodies [24].

The present cross-sectional study was, therefore, initiated to test the strength of the association between NT-proBNP and total ketone bodies in individuals with and without HF using data from the population-based Prevention of REnal and Vascular ENd-stage Disease (PREVEND) cohort study, which was conducted in the north of the Netherlands [10,25]. We also determined the extent to which such an association is modified by age, sex, prevalent atherosclerotic CVD, diabetes and obesity, and to what extent this may be different in individuals with and without HF.

## 2. Patients and Methods

### 2.1. Study Population and Definitions

The PREVEND study is a population-based cohort study carried out in the city of Groningen, the Netherlands. The design of the PREVEND Study has been described in detail elsewhere [25]. Briefly, from 1997 to 1998, all residents from Groningen, excluding pregnant women and people with type 1 diabetes or type 2 diabetes (T2D) using insulin, aged 28–75 years were invited to participate; a total of 8592 individuals completed an extensive examination [25]. For the present analysis, data from participants who completed the second screening round (2001–2003; n = 6894) were used. We excluded participants with missing data on HF and those who were not fasting. Participants with missing total ketone bodies and NT proBNP measurements were also excluded. This resulted in a study population of 6217 participants. The PREVEND study was approved by the Institutional Review Board of the University Medical Center Groningen (Medisch Ethische Toetsingscommissie (METc), IRB no. 01/139, approval date: 25 March 1996), and conforms with the Declaration of Helsinki. All participants provided written informed consent.

Heart failure (HF) was diagnosed using criteria described in the HF Guidelines of the European Society of Cardiology as published in 2012 and as applied previously [10]. HF was diagnosed if patients had presented with symptoms of breathlessness, ankle swelling, fatigue, orthopnea and paroxysmal nocturnal dyspnoea and/or clinical signs (i.e., elevated jugular venous pressure, pulmonary crackles and displaced apex beat), combined with objective measures of cardiac dysfunction [26]. Based on left ventricular ejection fraction (LVEF) at the time of diagnosis, HFrEF was categorized if LVEF ≤40% [27]. The rest was categorized as HFpEF. LVEF data were available through imaging techniques, such as echocardiography, magnetic resonance imaging (MRI) or radionuclide ventriculography [27]. Prevalent T2D was ascertained considering one or more of the following criteria: (1) fasting plasma glucose (FPG) ≥ 7.0 mmol/L (126 mg/dL); (2) random sample plasma glucose ≥ 11.1 mmol/L (200 mg/dL); (3) self-report of a physician diagnosis of T2D and (4) use of glucose-lowering medication, as retrieved from a central pharmacy registry.

During two outpatient visits (on two consecutive days), baseline data were collected on demographics, anthropometric measurements and medical history. Participants were studied after an overnight fast. Systolic and diastolic blood pressure values were recorded as the means of the last two recordings of the second visit, using an automatic Dinamap XL Model 9300 series device. Urinary albumin excretion (UAE) was measured in a 24 hr urine collection. Body mass index (BMI) was calculated as body weight divided by height squared (kg/m^2^). Waist circumference was measured on bare skin between the tenth rib and the iliac crest (cm). The waist–hip ratio was calculated as the ration between waist and hip circumference. Venous blood was taken after 15 min of rest. The estimated glomerular filtration rate (eGFR) was calculated using the combined creatinine-cystatin C equation of the Chronic Kidney Disease Epidemiology Collaboration (CKD-EPI) [28].

### 2.2. Laboratory Methods

Ethylene diamine tetraacetic acid (EDTA)-anticoagulated plasma samples were collected on ice and aliquots were frozen at −80 °C until analysis. N-terminal pro-B-type natriuretic peptide (NT-proBNP) measurements were performed on plasma using an ElecsysTM 2010 analyzer, a commercially available electrochemiluminescent sandwich immunoassay (Elecsys proBNP, Roche Diagnostics, Mannheim, Germany). The intra- and inter-assay coefficient of variation were 1.2–1.5 and 4.4–5.0%, respectively, with an analytical range of 5–35,000 ng/L. The conversion of NT-proBNP levels were as follows: 100 ng/L equates to 11.82 pmol/L. Plasma samples for measurement of ketone bodies were sent frozen to Labcorp, Morrisville, NC, USA for analysis. Total ketone bodies were calculated as the sum of β-hydroxybutyrate, acetoacetate and acetone, measured using a Vantera^®^ Clinical Analyzer (Labcorp, Morrisville, NC, USA), a fully automated, high-throughput, 400 MHz proton (1H) nuclear magnetic resonance (NMR) spectroscopy platform, as detailed elsewhere [10,29]. Quantification by NMR was compared to platforms commonly used for determining ketone body concentrations, that is, liquid chromatography/mass chromatography/mass chromatography (LC/MS/MS) for β-hydroxybutyrate and acetoacetate and gas chromatography/mass spectrometry (GC/MS) for acetone. Plasma concentrations found by NMR using the comparator platforms correlated well by Deming regression with R^2^ values of 0.996, 0.994 and 0.994 for β-hydroxybutyrate, acetoacetate and acetone, respectively. For β-hydroxybutyrate, acetoacetate and acetone, coefficients of variation for intra-assay and inter-assay precision were 1.3–9.3%, 3.1–7.7% and 3.8–9.1%, respectively. The urinary albumin concentration was determined by nephelometry, with a threshold of 2.3 mg/L and an intra- and inter-assay coefficient variation of 2.2–2.6%, respectively (BNII, Dade Behring Diagnostica, Marburg, Germany). FPG and total cholesterol (TC) were measured by dry chemistry (Eastman Kodak, Rochester, NY, USA). Triglycerides (TG) were analyzed on a Mega multi-analyzer (GPO PAP, Merck, Darmstadt, Germany). Serum creatinine was determined by conducting enzymatic measurement on a Roche Modular analyzer (Roche Diagnostics, Mannheim, Germany). Cystatin C was measured with a particle-enhanced turbidimetric immunoassay (Cystatin C PETIA assay, Gentian, Moss Norway) on a Roche auto-analyzer (Almere, The Netherlands).

### 2.3. Statistical Analysis

Statistical analyses were performed using STATA version 17 (StataCorp., 2021, Stata Statistical Software: Release 17; College Station, TX, USA: StataCorp LLC). Results were expressed as mean (standard deviation), median (interquartile range) or numbers (n) (percentage) for normally distributed, skewed and categorical data, respectively. Comparisons of baseline characteristics and circulating ketone bodies between participants with and without HF were tested using an independent sample *t* test, a Mann–Whitney U test or a chi-square test where appropriate. Pearson correlation coefficients were calculated to establish univariable relationships between variables. Multivariable linear regression analyses were carried out to disclose the independent association of plasma ketone bodies with NT-proBNP. In these analyses, to meet the assumption of normal distribution, ketone bodies, NT-proBNP, TG and urinary albumin excretion rates were log10 transformed. Interaction terms were calculated as the product terms of NT-proBNP with the variables of interest. A two-sided *p*-value < 0.05 was considered to indicate statistical significance.

## 3. Results

6217 participants were included in the study. Of these, 203 participants were diagnosed with HF; 6014 participants did not have HF. Participants with HF were older, more likely to be men, more (centrally) obese, more likely to have a positive history of CVD and to have T2D (Table 1). Systolic and diastolic blood pressure was higher in participants with HF. Participants with HF used angiotension-converting enzyme (ACE) inhibitors, angiotensin receptor blockers (ARBs), betablockers, diuretics and statins more frequently. Participants with HF had approximately four-fold higher levels of NT-proBNP. Total ketone bodies were approximately 25% higher in HF participants, which was attributable to increases in β-hydroxybutyrate, acetoacetate and acetone (Table 1). They also had lower TC, higher TG, lower eGFR and higher urinary albumin excretion (Table 1).

Of the 203 HF participants, 146 were categorized with HFrEF and 57 with HFpEF (Table 2). Participants with HFpEF were more likely to be women, had a higher BMI and tended to be more frequently diagnosed with T2D. NT-proBNP levels were higher in participants with HFrEF, but total ketone body levels were similar in participants with HFrEF and HFpEF (Table 2).

**Figure 1:**
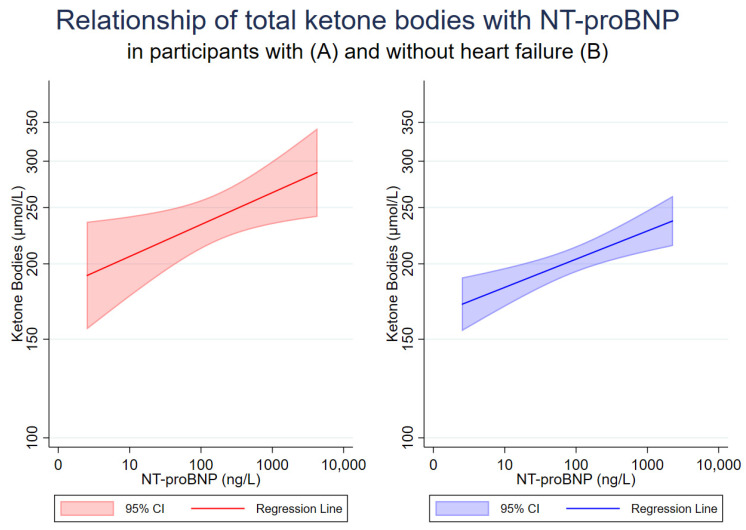
Relationship of total ketone bodies with NT-proBNP in participants with (**A**) and without (**B**) heart failure. Univariable linear association of log-transformed total ketone bodies and log-transformed NT-proBNP with corresponding 95% confidence intervals.

Univariable correlation analysis showed that in the whole cohort (Table 3A) and in participants without HF (Table 3C), total ketone body levels were positively correlated with age, BMI, waist circumference, waist–hip ratio, systolic and diastolic blood pressure, history of T2D, use of ACE inhibitors, ARBs (in whole cohort) and UAE and inversely with eGFR. In these groups, total ketone body levels, as well as β-hydroxybutyrate and acetoacetate, were positively correlated with NT-proBNP (Table 3A,C). In participants with HF, as well as in participants with HFrEF, there was also a positive association of total ketone bodies with NT-proBNP and, in addition, with TG (Table 3B,D). In participants with HFpEF, the association of total ketone bodies with NT-proBNP did not reach statistical significance, although total ketone body levels were inversely associated with BMI and waist circumference, and positively associated with TG (Table 3E). Figure 1 shows the univariable relationship of total ketone bodies with NT-proBNP among participants with and without HF.

In multivariable analysis in the whole cohort and among participants without HF, total ketone bodies, as well as β-hydroxybutyrate and acetoacetate separately, were associated with NT-proBNP, both in crude and in age- and sex-adjusted analyses (Table 4, Model 1). This association was not materially attenuated after further adjustment for eGFR, UAE, systolic blood pressure, T2D and CVD history (Model 2), and remained similar after additional adjustment for either BMI (Model 3), waist circumference (Model 4) or waist–hip ratio (Model 5). Similarly, total ketone bodies, β-hydroxybutyrate and acetone were associated with NT-proBNP in participants with HF in age- and sex-adjusted analyses (Table 4B). β-hydroxybutyrate was significantly associated with NT-proBNP in both HF and HFrEF and remained statistically significant after multivariable adjustment. In participants with HFpEF, these associations did not attain statistical significance (Table 4E).

**Figure 2:**
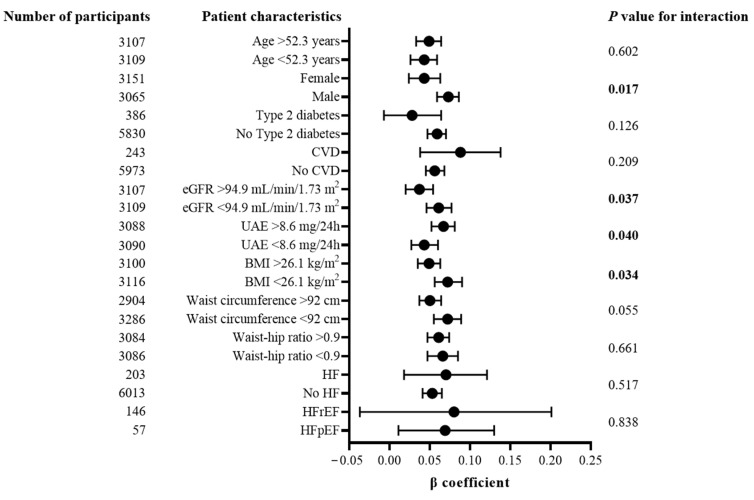
Regression coefficients of linear regression of NT-proBNP on total ketone bodies, by relevant participant-level characteristics. NT-proBNP: N-terminal pro-B-type natriuretic peptide; BMI: body mass index; CVD: cardiovascular disease; eGFR: estimated glomerular filtration rate; UAE: urinary albumin excretion. Total ketone bodies, NT-proBNP and UAE were log transformed. Statistically significant interactions are indicated in bold print.

In exploring interactions of NT-proBNP with clinical variables and laboratory variables impacting total ketone bodies, NT-proBNP interacted with sex, eGFR, UAE and BMI in such a way that these associations were stronger in males, participants with lower eGFR, higher UAE and lower BMI. There were no significant interactions with waist circumference and waist–hip ratio. Notably, there were also no interactions with respect to the presence of HF, nor between HFrEF and HFpEF (Figure 2).

## 4. Discussion

The present general population-based study has demonstrated that fasting ketone bodies are elevated in individuals with HF together with anticipated increases in NT-proBNP. Notably, we documented a positive association of fasting circulating total ketone bodies with NT-proBNP. Such an association was also found for β-hydroxybutyrate and acetoacetate separately, and remained statistically significant after adjustment for age, sex, prevalent diabetes and a history of CVD, eGFR, urinary albumin excretion, systolic blood pressure and obesity measures (i.e., BMI, waist circumference or waist–hip ratio). There was an interaction of sex, eGFR, UAE and BMI impacting the association of total ketone bodies with NT-proBNP, suggesting that this relationship was stronger in male participants with compromised kidney function and in less obese individuals. Importantly, there was no interaction with the presence of HF. Together, our epidemiological findings agree with the possibility that circulating NT-proBNP could be involved in the metabolic regulation of plasma ketone bodies [18,19,20,21,22]. We suggest that an increase in circulating ketone bodies in HF could conceivably provide alternative energy substrates for a jeopardized myocardium, constituting part of a metabolic defense mechanism [12,13,14].

The present findings regarding the elevations in circulating ketone bodies in participants with HF are in line with earlier observations [7,8]. The highest NT-proBNP levels were found in participants with HFrEF, in whom BMI, waist circumference and diabetes prevalence tended to be lower compared to those with HFpEF [29,30]. The modest association of ketone bodies with NT-proBNP warrants a comparison with a Japanese study conducted on a high-risk CVD population subjected to cardiac catheterization [24]. Within this study’s population, 74% had ischemic heart disease and 7.9% had cardiomyopathy as underlying pathologies; 81% were men. The participants of that study were not evaluated after a standardized fasting period. BNP, rather than NT-proBNP, was measured and total ketone body levels were assayed using an enzymatic method. Plasma total ketone bodies amounted to 194 µmol/L, similar to the values obtained in the current report. In the Japanese study, left ventricular end-diastolic pressure, left ventricular end-systolic volume index and left ventricular end-diastolic volume index were unrelated to ketone bodies. There was a stronger univariable correlation of BNP with ketone bodies than that which we found in our population-based study. Remarkably, there was also a strong inverse correlation of ketone bodies with TG despite a modest positive correlation with BMI, an unusual finding given the close positive relationship between obesity and TG [31]. This observation may reflect the non-standardized nutritional status of the study participants, as individuals who have recently consumed a meal are more inclined to exhibit elevated TG levels while concurrently displaying reduced levels of ketone bodies. We found a modest correlation of ketone bodies with BMI, waist circumference, waist–hip ratio and TG, at least in the whole cohort and in participants without HF. The association of NT-proBNP with ketone bodies was found to be more pronounced in individuals with a lower BMI. This may be due to a lower expression of NP receptors in obese individuals, resulting in less lipolysis in response to NPs, and therefore less substrate availability for ketogenesis [32]. However, in another report involving PREVEND participants, ketone bodies were increased, but NT-proBNP was decreased in subjects with suspected non-alcoholic fatty liver disease (NAFLD) (current nomenclature metabolic associated steatotic liver disease or MASLD) [33]. As NAFLD was estimated in that study by the fatty liver index (FLI), an algorithm that relies on BMI and waist circumference [34], this suggests that the association of ketone bodies with NT-proBNP is unlikely to be significantly influenced by obesity. In line with this, the association of ketone bodies with NT-proBNP was not mitigated after adjustment for either BMI or waist circumference in the current study, although the association of total ketone bodies with NT-proBNP was more pronounced in participants with a lower BMI.

The association between NT-proBNP and total ketone bodies was not different between participants with or without HF, nor between individuals with HFrEF vs. HFpEF, as reflected by the lack of significant interactions of NT-proBNP impacting circulating total ketone bodies. This suggests that with higher NT-proBNP concentrations, as found in participants with HF, circulating total ketone bodies are similarly higher as compared to participants without HF. Assuming that the uptake of ketone bodies in the human myocardium is proportional to their plasma concentrations [13], this would suggest that the heart consumes more ketone bodies in HF, a phenomenon which could be enhanced in the context of compromised kidney function. In HF, multiple metabolic changes occur that include enhanced uptake and oxidation of ketone bodies, in particular, β-hydroxybutyrate [35]. Moreover, though yet to be understood in more detail, β-hydroxybutyrate may improve cardiac inflammatory responses and inhibit mitochondrial oxidative stress through multiple mechanisms [14].

The association between ketone bodies and NT-proBNP may also have relevance for a better understanding of the underlying pathophysiology, whereby sodium-dependent glucose-cotransporter protein 2 (SGLT2) inhibitors improve cardiovascular outcomes in patients with HFrEF and HFpEF and in patients with recent worsening or acute heart failure [23,36,37,38,39,40]. SGLT2 inhibitors are known to induce ketosis, which may provide a mechanism for cardiac benefit [14,41,42,43,44]. This effect of SGLT2 inhibitors to increase ketone bodies has been attributed to a decrease in blood glucose levels [42,43]. However, SGLT2 inhibitors may also enhance lipolysis via catecholamine-induced stimulation of β3-adrenoceptors in adipocytes, which activate hormone sensitive lipase via a cyclic adenosine monophosphate (cAMP)-dependent cascade of events [45]. In contrast, NPs stimulate lipolysis via a NP receptor typeA/cyclic guanosine monophosphate (cGMP)-dependent pathway, independent of catecholamine action and cAMP production [21,22,46]. In this regard, it is remarkable that in patients with acute worsening of heart failure, there were no differences in plasma ketone body levels between those treated with SGLT2 inhibitors and those treated with the placebo [23]. We surmise that during high levels of systemic stress, as in acute heart failure, catecholamine stimulation of lipolysis is enhanced, obviating further elevations in circulating ketone bodies elicited by high circulating NPs.

Finally, we have focused on potential beneficial effects of ketone bodies with respect to fuel delivery to the failing heart. Nonetheless, it is important to note that higher plasma total ketone body concentrations, as well as β-hydroxybutyrate, acetoacetate and acetone separately, were found to be associated with increased all-cause mortality, even when taking NT-proBNP into account [33]. In that study, the impact of (suspected) NAFLD on mortality was in part mediated by ketone body levels [33], a relevant finding because obesity is a strong risk factor for both NAFLD and HF [47,48,49]. Moreover, plasma ketone bodies are associated with new-onset diabetes in individuals with normal fasting glucose initially [50], whereas higher ketone bodies relate to worse glycemic control among patients with established T2D [51].

The current study has strengths and limitations. The well-documented nature of the PREVEND cohort and its large sample size may be considered a strength. Its descriptive nature and cross-sectional design precludes the ability to establish causality. Moreover, neither the plasma insulin/glucagon ratio nor plasma catecholamines were measured in the present study, obviating the ability to compare the strength of the association of these measures with plasma ketone bodies with that of NT-proBNP. Furthermore, the analysis within the subgroup of participants with HFpEF might be underpowered due to the rather small number of participants with HFpEF. Therefore, conclusions on this specific subgroup are only possible to a limited extent. No deductions can be made on the association of ketone bodies with the EF in a continuous manner, as data on exact EF values were not documented. The classification into HF type was conducted using the ESC 2012 guidelines; therefore, no patients were classified as having HF with mid-range ejection fraction (HFmrEF). Due to the data collection in 2001–2003, the influence of current HF guideline-recommended medications, such as sodium-glucose cotransporter-2 inhibitors, on the association of ketone bodies with NT-proBNP is not addressed. The voluntary inclusion process of the PREVEND study may result in a selection of participants in relatively good health. This could possibly involve inclusion of milder cases of HF, as reflected by comparatively low NT-proBNP levels. The PREVEND cohort study consists of primarily Dutch patients of white ethnicity, which could limit the generalizability of these findings to other regions and ethnicities.

## 5. Conclusions

The current study is the first to show an association of fasting total body ketone bodies, as well as β-hydroxybutyrate and acetoacetate, with plasma NT-proBNP in a large general population which remained after adjustment for relevant covariates. This association was not modified by the presence of HF. With both plasma NT-proBNP and ketone bodies being elevated in participants with HF, our findings lend support to the notion that a metabolic defense mechanism may be operative, providing the myocardium with ketone bodies to cope with the demands for fuel delivery.

## Figures and Tables

**Table 1:** Characteristics of participants with and without heart failure (HF).

	All Participants	Participants with HF	Participants without HF	*p*-Value
Number	6217	203	6014	
Age, years	53.6 (12.0)	65.87 (9.9)	53.1 (11.9)	<0.001
Females, n (%)	3149 (50.7%)	66 (32.5%)	3083 (51.3%)	<0.001
BMI, kg/m^2^	26.69 (4.35)	28.79 (4.49)	26.62 (4.33)	<0.001
Waist circumference, cm	92.1 (12.8)	101.4 (13.4)	91.7 (12.7)	<0.001
Waist–hip ratio	0.90 (0.09)	0.96 (0.09)	0.90 (0.09)	<0.001
SBP, mmHg	126 (19)	138 (24)	126 (18)	<0.001
DBP, mmHg	73 (9)	76 (10)	73 (9)	<0.001
CVD, n (%)	243 (3.9%)	49 (24.1%)	194 (3.2%)	<0.001
Type 2 diabetes, n (%)	386 (6.2%)	44 (21.7%)	342 (5.7%)	<0.001
Hypertension, n (%)	654 (10.7%)	65 (32.7%)	589 (10.0%)	<0.001
Total cholesterol, mmol/L	5.43 (1.05)	5.28 (1.06)	5.43 (1.05)	0.036
Triglycerides, mmol/L	1.11 (0.81, 1.61)	1.28 (1.00, 1.71)	1.11 (0.80, 1.61)	<0.001
Plasma NT-proBNP, ng/L	42 (21, 81)	168 (75, 443)	41 (21, 77)	<0.001
β-hydroxybutyrate, µmol/L	122.0 (93.2, 170.1)	153.9 (111.3, 237.3)	121.3 (92.8, 168.8)	<0.001
Acetoacetate, µmol/L	38.4 (25.8, 57.4)	46.58 (29.2, 74.8)	38.18 (25.7, 57.0)	<0.001
Acetone, µmol/L	19.6 (12.7, 29.2)	24.24 (16.0, 36.3)	19.5 (12.6, 29.0)	<0.001
Total ketone bodies, µmol/L	177.8 (139.2, 250.2)	218.7 (162.5, 338.0)	176.7 (138.8, 247.4)	<0.001
eGFR, mL/min/1.73 m^2^	92.9 (16.9)	76.11 (20.0)	93.43 (16.5)	<0.001
UAE, mg/24h	8.6 (6.0, 16.1)	16.4 (8.0, 59.8)	8.5 (6.0, 15.5)	<0.001
ACEi use, n (%)	383 (6.5%)	48 (23.9%)	335 (5.9%)	<0.001
ARB use, n (%)	127 (2.1%)	24 (11.9%)	103 (1.8%)	<0.001
Betablocker use, n (%)	594 (10.0%)	68 (33.8%)	526 (9.2%)	<0.001
Statin use, n (%)	474 (8.0%)	53 (26.4%)	421 (7.4%)	<0.001
Diuretic use, n (%)	214 (3.6%)	30 (14.9%)	184 (3.2%)	<0.001

Values are displayed as mean (±SD), median (interquartile range) or n (%). BMI: body mass index; SBP: systolic blood pressure; DBP: diastolic blood pressure; CVD: cardiovascular disease; NT-proBNP: N-terminal pro-B-type natriuretic peptide; eGFR: estimated glomerular filtration rate; UAE: urinary albumin excretion; ACEi: angiotensin-converting enzyme inhibitor; ARB: angiotensin receptor blocker.

**Table 2:** Characteristics of participants with heart failure with reduced ejection fraction (HFrEF) and preserved ejection fraction (HFpEF).

	HFrEF	HFpEF	*p*-Value
Number	146	57	
Age, years	65.22 (10.24)	67.52 (8.62)	0.130
Females, n (%)	38 (26.0%)	28 (49.1%)	0.002
BMI, kg/m^2^	28.2 (4.3)	30.2 (4.6)	0.004
Waist circumference, cm	101.0 (13.3)	102.7 (13.9)	0.390
Waist–hip ratio	0.96 (0.08)	0.94 (0.10)	0.110
SBP, mmHg	136 (21)	144 (30)	0.041
DBP, mmHg	76 (10)	76 (11)	0.700
CVD, n (%)	40 (27.4%)	11 (19.3%)	0.230
Type 2 diabetes, n (%)	28 (19.2%)	16 (28.1%)	0.170
Hypertension, n (%)	45 (31.3%)	20 (36.4%)	0.490
Total cholesterol, mmol/L	5.23 (1.09)	5.39 (0.96)	0.340
Triglycerides, mmol/L	112.1 (88.4, 157.9)	115.4 (92.1, 134.4)	0.970
Plasma NT-proBNP, ng/L	192 (90, 548)	112 (68, 237)	0.0320
β-hydroxybutyrate, µmol/L	150.3 (113.8, 237.3)	156.5 (111.3, 233.6)	0.890
Acetoacetate, µmol/L	46.5 (29.1, 74.8)	46.6 (29.8, 74.8)	0.890
Acetone, µmol/L	24.2 (15.39, 35.2)	24.39 (16.8, 38.1)	0.440
Total ketone bodies, µmol/L	217.9 (164.6, 329.3)	230.2 (157.8, 372.8)	0.950
eGFR, mL/min/1.73 m^2^	76.6 (20.3)	74.8 (19.5)	0.560
UAE, mg/24h	17.0 (8.0, 59.8)	14.38 (9.1, 54.6)	0.940
ACEi use, n (%)	36 (24.8%)	12 (21.4%)	0.610
ARB use, n (%)	17 (11.7%)	7 (12.5%)	0.880
Betablocker use, n (%)	46 (31.7%)	22 (39.3%)	0.310
Statin use, n (%)	42 (29.0%)	11 (19.6%)	0.180
Diuretic use, n (%)	19 (13.1%)	11 (19.6%)	0.240

Values are displayed as mean (±SD), median (interquartile range) or n (%). BMI: body mass index; SBP: systolic blood pressure; DBP: diastolic blood pressure; CVD: cardiovascular disease; NT-proBNP: N-terminal pro-B-type natriuretic peptide; eGFR: estimated glomerular filtration rate; UAE: urinary albumin excretion; ACEi: angiotensin-converting enzyme inhibitor; ARB: angiotensin receptor blocker.

**Table 3:** Pearson correlation coefficients of total ketone bodies with clinical and laboratory characteristics in **A.** all participants (N = 6217); **B.** in participants with heart failure (N = 203); **C.** in participants without heart failure (N = 6014); **D.** participants with heart failure with a reduced ejection fraction. (N = 146); **E.** participants with heart failure with a preserved ejection fraction (N = 57).

	(A) All Participants	*p*	(B) Participants with HF	*p*	(C) Participants without HF	*p*	(D) Participants with HFrEF	*p*	(E) Participants with HFpEF	*p*
Age	**0.167**	**<0.001**	0.122	0.122	**0.158**	**<0.001**	0.125	0.133	0.051	0.706
Female sex	−0.004	0.758	0.061	0.385	−0.001	0.923	0.086	0.304	−0.014	0.920
BMI	**0.074**	**0.012**	−0.019	0.788	**0.071**	**<0.001**	0.076	0.365	**−0.263**	**0.048**
Waist circumference	**0.090**	**<0.001**	−0.012	0.865	**0.085**	**<0.001**	0.071	0.399	−0.223	0.098
Waist–hip ratio	**0.072**	**<0.001**	−0.041	0.565	**0.068**	**<0.001**	0.024	0.779	−0.174	0.199
SBP	**0.123**	**<0.001**	0.105	0.138	**0.116**	**<0.001**	0.149	0.074	−0.027	0.840
DBP	**0.077**	**<0.001**	0.004	0.958	**0.076**	**<0.001**	0.048	0.569	0.018	0.896
CVD	**0.035**	**0.006**	0.047	0.505	0.020	0.127	−0.011	0.897	0.225	0.092
Type 2 diabetes	**0.130**	**<0.001**	**0.184**	**0.009**	**0.119**	**<0.001**	0.162	0.051	0.221	0.098
Total cholesterol	−0.002	0.856	−0.075	0.293	0.002	0.859	−0.082	0.328	−0.066	0.753
Triglycerides	**0.047**	**<0.001**	**0.163**	**0.020**	**0.041**	**0.001**	**0.211**	**0.011**	0.045	0.736
NT-proBNP	**0.133**	**<0.001**	**0.185**	**0.008**	**0.116**	**<0.001**	**0.195**	**0.019**	0.182	0.173
eGFR	**−0.135**	**<0.001**	**−0.184**	**0.009**	**−0.121**	**<0.001**	**−0.213**	**0.010**	−0.106	0.430
UAE	**0.110**	**<0.001**	0.129	0.067	**0.100**	**<0.001**	0.132	0.112	0.124	0.356
ACEi use	**0.062**	**<0.001**	−0.015	0.835	**0.058**	**<0.001**	−0.012	0.890	−0.017	0.897
ARB use	**0.033**	**0.011**	0.058	0.415	0.021	0.107	0.109	0.192	−0.064	0.640
Beta-blocker use	**0.034**	**0.009**	−0.006	0.928	0.025	0.058	−0.016	0.853	0.004	0.971
Statin use	**0.063**	**<0.001**	−0.012	0.869	**0.059**	**<0.001**	0.016	0.849	−0.074	0.589
Diuretic use	**0.039**	**0.002**	−0.002	0.979	**0.034**	**0.009**	0.016	0.848	−0.048	0.725

BMI: body mass index; SBP: systolic blood pressure; DBP: diastolic blood pressure; CVD: cardiovascular disease; NT-proBNP: N-terminal pro-B-type natriuretic peptide; eGFR: estimated glomerular filtration rate; UAE: urinary albumin excretion; ACEi: angiotensin-converting enzyme inhibitor; ARB: angiotensin receptor blocker. Triglycerides, ketone bodies, NT-proBNP and UAE are log transformed. Statistically significant correlations are indicated in bold print.

**Table 4:** Multivariable linear regression analysis on total ketone bodies, β-hydroxybutyrate, acetoacetate and acetone with NT-proBNP in all participants (N = 6217), participants with heart failure (HF) (N = 203), participants without heart failure (N = 6014), participants with heart failure with reduced ejection fraction (HFrEF) (N = 146)**,** participants with heart failure with preserved ejection fraction (HFpEF) (N = 57). Model 1: age and sex adjusted. Model 2: age, sex, eGFR, UAE, systolic blood pressure and CVD adjusted. Model 3: age, sex, eGFR, UAE, systolic blood pressure, CVD and BMI adjusted. Model 4: age, sex, eGFR, UAE, systolic blood pressure, CVD and waist-circumference adjusted. Model 5: age, sex, eGFR, UAE, systolic blood pressure, CVD and waist–hip ratio adjusted.

	Total Ketone Bodies		β-Hydroxybutyrate		Acetoacetate		Acetone	
	Std β	*p*-Value	Std β	*p*-Value	Std β	*p*-Value	Std β	*p*-Value
(A) All participants								
Crude	**0.13**	**<0.001**	**0.16**	**<0.001**	**0.07**	**<0.001**	0.01	0.318
Model 1	**0.08**	**<0.001**	**0.09**	**<0.001**	**0.05**	**0.002**	0.01	0.357
Model 2	**0.08**	**<0.001**	**0.09**	**<0.001**	**0.05**	**0.002**	0.01	0.683
Model 3	**0.08**	**<0.001**	**0.09**	**<0.001**	**0.05**	**0.002**	0.01	0.637
Model 4	**0.08**	**<0.001**	**0.09**	**<0.001**	**0.05**	**0.002**	0.01	0.611
Model 5	**0.08**	**<0.001**	**0.09**	**<0.001**	**0.05**	**0.002**	0.01	0.627
(B) Participants with HF								
Crude	**0.19**	**0.008**	**0.20**	**0.005**	0.12	0.098	**0.17**	**0.019**
Model 1	**0.17**	**0.024**	**0.19**	**0.014**	0.09	0.224	**0.15**	**0.042**
Model 2	0.15	0.060	**0.17**	**0.036**	0.08	0.365	0.16	0.062
Model 3	0.15	0.078	**0.16**	**0.047**	0.06	0.436	0.15	0.076
Model 4	0.16	0.052	**0.18**	**0.030**	0.08	0.378	0.16	0.056
Model 5	**0.16**	**0.049**	**0.18**	**0.030**	0.08	0.357	**0.17**	**0.047**
(C) Participants without HF								
Crude	**0.12**	**<0.001**	**0.15**	**<0.001**	**0.06**	**<0.001**	−0.01	0.502
Model 1	**0.07**	**<0.001**	**0.09**	**<0.001**	**0.04**	**0.014**	−0.01	0.930
Model 2	**0.07**	**<0.001**	**0.08**	**<0.001**	**0.04**	**0.008**	−0.01	0.691
Model 3	**0.07**	**<0.001**	**0.08**	**<0.001**	**0.04**	**0.007**	−0.01	0.750
Model 4	**0.07**	**<0.001**	**0.08**	**<0.001**	**0.04**	**0.006**	−0.01	0.793
Model 5	**0.07**	**<0.001**	**0.08**	**<0.001**	**0.04**	**0.006**	−0.01	0.765
(D) Participants with HFrEF								
Crude	**0.19**	**0.019**	**0.21**	**0.011**	0.10	0.249	**0.19**	**0.020**
Model 1	0.16	0.068	**0.18**	**0.043**	0.05	0.569	0.18	0.050
Model 2	0.17	0.086	**0.19**	**0.049**	0.05	0.610	0.18	0.069
Model 3	0.18	0.063	**0.21**	**0.032**	0.06	0.585	0.19	0.057
Model 4	0.19	0.052	**0.22**	**0.025**	0.06	0.557	0.19	0.061
Model 5	0.18	0.060	**0.21**	**0.032**	0.06	0.542	0.19	0.066
(E) Participants with HFpEF								
Crude	0.18	0.173	0.18	0.170	0.18	0.192	0.11	0.412
Model 1	0.19	0.195	0.20	0.168	0.17	0.244	0.11	0.478
Model 2	0.07	0.725	0.08	0.660	0.06	0.774	0.03	0.861
Model 3	0.12	0.513	0.13	0.462	0.09	0.619	0.08	0.680
Model 4	0.12	0.530	0.13	0.473	0.10	0.615	0.06	0.752
Model 5	0.05	0.802	0.06	0.746	0.03	0.882	0.06	0.779

Std β: standardized regression coefficients. BMI body mass index; SBP: systolic blood pressure; CVD: cardiovascular disease; NT-proBNP: N-terminal pro-B-type natriuretic peptide; eGFR: estimated glomerular filtration rate; UAE: urinary albumin excretion; T2D: type 2 diabetes; CVD: cardiovascular disease. Total ketone bodies, β-hydroxybutyrate, acetoacetate, acetone, NT-proBNP and UAE are log transformed. Statistically significant correlations are indicated in bold print.

## Data Availability

The data presented in this study are available on request from the corresponding author. The data are not publicly available due to privacy.
